# Protein modification by short-chain fatty acid metabolites in sepsis: a comprehensive review

**DOI:** 10.3389/fimmu.2023.1171834

**Published:** 2023-10-06

**Authors:** Liang Zhang, Xinhui Shi, Hongmei Qiu, Sijia Liu, Ting Yang, Xiaoli Li, Xin Liu

**Affiliations:** ^1^ Department of Pharmacology, College of Pharmacy, Chongqing Medical University, Chongqing, China; ^2^ Chongqing Key Laboratory of Drug Metabolism, Chongqing, China; ^3^ Key Laboratory for Biochemistry and Molecular Pharmacology of Chongqing, Chongqing, China; ^4^ Medical Research Center, Southwest Hospital, Third Military Medical University, Chongqing, China

**Keywords:** sepsis, short-chain fatty acids (SCFAs), protein post-translational modifications (PTMs), immunity, inflammation

## Abstract

Sepsis is a major life-threatening syndrome of organ dysfunction caused by a dysregulated host response due to infection. Dysregulated immunometabolism is fundamental to the onset of sepsis. Particularly, short-chain fatty acids (SCFAs) are gut microbes derived metabolites serving to drive the communication between gut microbes and the immune system, thereby exerting a profound influence on the pathophysiology of sepsis. Protein post-translational modifications (PTMs) have emerged as key players in shaping protein function, offering novel insights into the intricate connections between metabolism and phenotype regulation that characterize sepsis. Accumulating evidence from recent studies suggests that SCFAs can mediate various PTM-dependent mechanisms, modulating protein activity and influencing cellular signaling events in sepsis. This comprehensive review discusses the roles of SCFAs metabolism in sepsis associated inflammatory and immunosuppressive disorders while highlights recent advancements in SCFAs-mediated lysine acylation modifications, such as substrate supplement and enzyme regulation, which may provide new pharmacological targets for the treatment of sepsis.

## 1 Introduction

Sepsis, according to the latest criteria (Sepsis 3.0) is defined a life-threatening organ dysfunction syndrome caused by a dysregulated host response due to infection or infectious agents. It serves as one of the most common complications in patients with clinical trauma/burns and infections while ranks a leading cause of death in critically ill units (1). An estimated number of 31.5 million cases and 5.3 million deaths are recorded worldwide in a single year, making sepsis as a prominent health problem for the global medical community (2). Sepsis is traditionally recognized as a two- stage syndrome which manifests with hyperinflammation and immune suppression. More recently, it is suggested that sepsis may manifest concurrent hyperinflammation and immune suppression (3). In recent years, substantial progress has been made in revealing the mechanisms that drives the development of sepsis, especially in the areas of metabolisms and epigenetics (4). Remarkably, profound alterations in intracellular metabolites and protein epigenetic markers have been identified as key regulatory mechanisms for the initiation of inflammation and immunosuppression as well as their phenotypic transformation. These findings hold significant implications for understanding the immunopathology of sepsis and the search for potential drug intervention targets.

The primary events of sepsis include infection, inflammation induced by infection, immune dysregulation, and organ dysfunction. Sepsis also involves various events that are secondary to those primary changes. During sepsis, notably changes in host metabolism have been detected, such as hyperglycemia, aberrant lipid metabolism, and amino acid metabolism disorders, leading to significant alterations in the level and activity of cellular metabolites that result in immune dysfunction (5, 6). The modified levels of metabolites can affect the pathophysiological process of sepsis by either affecting the activity of enzymes or otherwise altering protein structures and interactions (7, 8). The protein post-translational modifications (PTMs), as a result of an altered metabolism, represent one of the main causes of functional diversity of mammalian protein molecules, and are thus involved in the regulation of protein function and activity by inducing covalent binding of proteins to different functional metabolites (such as acetate, butyrate and lactate) (9). The regulatory machinery of protein PTMs mainly include substrates, modifying enzymes and de-modifying enzymes, among which the substrates are directly derived from the transformation of cell-related metabolites (10, 11). For instance, lactate can serve as a substrate for lactylation, while acetyl-CoA can be utilized as a substrate for acetylation modification (12, 13). Additionally, certain metabolites act as endogenous inhibitors of chromatin-modifying enzymes, thereby influencing the levels of PTMs by modulating the activity of modifying and de-modifying enzymes (14, 15). For instance, butyrate is a metabolite that regulates PTMs by inhibiting histone deacetylase (HDAC) activity(16). Importantly, PTMs can occur in histones, affecting the regulation of gene expression, as well as in non-histone proteins, influencing their function or interactions with other proteins (17, 18). Histone modification includes methylation, acetylation, phosphorylation and other forms, which can regulate gene transcriptional activity and chromatin structure by changing the chemical structure of histone (19–21). The role of histone modification in the pathogenesis and development of sepsis is multifaceted, encompassing the regulation of inflammatory factor expression, modulation of immune cell function and differentiation, and regulation of cell apoptosis and survival (22–24). PTMs can also influence the activity of themselves or interactive proteins by acting on non-histone proteins, thereby regulating the level of autophagy and nuclear translocation that may intervene in the progression of sepsis (25, 26). Furthermore, metabolite-dependent PTMs may affect the short- and long-term immunosuppressive state by providing rapid and prolonged responses that counteract the overreaction of circulating acute pro-inflammatory cytokines in sepsis patients (27).

In recent years, the significance of intestinal flora and their metabolites in inflammatory and metabolism-related diseases has gained increasing recognition(28). Among these, short-chain fatty acids (SCFAs), obtained by the flora through fermentation of food fibers, have received considerable attention, particularly in their regulatory roles in metabolism, maintenance of the intestinal mucosal barrier, and immune homeostasis (29, 30). SCFAs and its corresponding acyl coenzyme A are located at the crossroads of metabolic pathways and play an important role in various cellular processes. It has been observed that SCFAs can play a signal transduction role through covalent or non-covalent binding to proteins and can also bind to modifying enzymes to mediate post-translational modification of proteins (31). Moreover, SCFAs have been found to exert major regulatory effects in sepsis, including affecting gene expression, enhancing phagocytosis of macrophages, altering cell proliferation and function, and inhibiting the activity of HDAC (32–36). Several recent studies have demonstrated that there were significant differences in SCFAs levels in the feces of severe septic patients and in murine models of sepsis induced by cecal ligation and puncture (CLP) (37, 38). Therefore, the disorder of SCFAs is secondary to changes in the primary event of sepsis, and changes in SCFAs levels may further affect the formation of PTMs, which in turn may mediate the primary event of sepsis.

In this review, we have provided a summary of the metabolic mechanisms involved in the inflammatory and immunosuppressive phases of sepsis, with a particular focus on the roles of SCFAs metabolism. We have also discussed recent advances in SCFAs-mediated lysine acylation modifications.

## 2 The intricate pathophysiology of sepsis and the underlying metabolic mechanisms

The pathogenesis of sepsis is a complex phenomenon, involving a multitude of factors spanning from the molecular to the organ level. This intricate interplay encompasses issues such as infection, inflammation, immunity, coagulation, and tissue damage (3). Cytokine storm is an early manifestation of sepsis, which primarily arises from macrophages engulfing pathogens and subsequently releasing a myriad of inflammatory cytokines, thereby triggering the body’s inflammatory response (39). In the later stages of sepsis, immunosuppression assumes a dominant role, characterized by the downregulation of pro-inflammatory factors, secondary infections, and increased apoptosis and autophagy of immune cells (40–42). Apoptosis of immune cells is a major factor in the development of immunosuppression in sepsis(43). Notably, sepsis is also suggested to manifest concurrent hyperinflammation and immune suppression that further increase the intricateness of this syndrome (3).

The alterations in the levels of relevant metabolites during sepsis can interfere with inflammatory and immune processes by regulating the levels of PTMs. Clinical investigations have shown that metabolic reprogramming during sepsis leads to profound changes in glucose, lipid, and fatty acid metabolism (44). Among these, glucose metabolic reprogramming occurs during sepsis when glycolytic pathways are enhanced in response to cellular biosynthesis and bioenergy requirements, thereby promoting cell growth, differentiation, and effector functions (45). Second, lipid production is an important adaptive response to normal tissue function. Clinical studies have found that cholesterol and lipoprotein levels vary markedly in patients with inflammation, with patients with severe sepsis having lower levels of cholesterol, including high-density lipoprotein (HDL), low-density lipoprotein (LDL) and apolipoprotein A-I (Apo A-I), as well as high levels of triglycerides (TGs) and free fatty acids (FFA) (46). Consequently, HDL and LDL are considered to be important modulators of the host immune response during sepsis. At the same time, sepsis induces the release of lipid mediators, many of which activate nuclear receptors. For instance, peroxisome proliferator-activated receptor (PPAR) α, a nuclear receptor activated by fatty acids (FAs), controls lipid metabolism and inflammation. Paumelle et al. found that PPARα deficiency led to a pro-inflammatory response and reduced survival in the CLP model, which impaired the adaptive metabolic shift from glucose to fatty acid (FAs) utilization (47).

Beyond the major metabolic pathways, some specific metabolic pathways and their products are also significantly altered in sepsis, such as SCFAs. During sepsis, increased inflammatory factors lead to intestinal epithelial cell apoptosis and increased intestinal wall permeability (48). In addition, sepsis induces kidney injury, with a sharp increase in urea, sodium, and water leading to intestinal wall edema and disruption of tight junction proteins in colonic epithelial cells. Inflammatory cytokines as well as urea retention damage the intestinal barrier, leading to bacterial translocation through the portal vein or mesenteric lymphatic system, causing a systemic inflammatory response and eventually leading to multiple organ failure and death (49, 50). Due to the effects of antibiotics, parenteral nutrition and systemic inflammation, the number of anaerobic bacteria in the intestinal flora is significantly down-regulated, accompanied by the significant down-regulation of intestinal metabolites SCFAs (48, 51). Studies have shown that SCFAs play an important role in immune regulation and inflammation suppression (52). For example, SCFAs can regulate the function of immune cells by activating G protein-coupled receptors, inhibit the release of inflammatory mediators, and mitigating the inflammatory response (53). SCFAs can also promote intestinal barrier function and regulate intestinal flora balance, potentially conferring effects against the occurrence and progression of sepsis (54). Furthermore, both murine models and *in vitro* experiments have demonstrated notable alterations in SCFAs levels within immune cells, strongly correlating with sepsis progression. For example, one study revealed fecal SCFAs levels were significantly downregulated in a rat sepsis model induced by CLP, while the levels of TNF-α, IL-1β, IL-6, and other inflammatory factors in the hippocampus were markedly upregulated (55). Additionally, exogenous supplementation of SCFAs in lipopolysaccharide (LPS)-stimulated primary rat neutrophils not only significantly suppressed the levels of TNF-α and nitric oxide synthase by inhibiting the activation of nuclear factor κB (NF-κB), but also inhibited the activity of HDAC, potentially influencing inflammation progression through the modulation of specific PTMs (56).

## 3 Gut microbiota and microbiota derived SCFAs

Gut microbiota refers to the community of various microorganisms present in the human gut, including bacteria, archaea, fungi, and viruses, plays a crucial role in human health (57). Recent studies have highlighted its involvement in various biological processes, such as the regulation of tumor cell immunogenicity and innate immune functions, particularly in tumor cells and in macrophages (58, 59). In addition, the interplay between the intestinal microbiota, local immunity, and gut integrity has emerged as a key determinant in disease development (60). During sepsis, factors like intestinal hypoperfusion, intestinal cell apoptosis, systemic cytokine storms, and intestinal dysbiosis contribute to the disruption of intestinal cell permeability, promoting the migration of flora and the transfer of inflammatory mediators, leading to multiple organ dysfunction syndrome and systemic inflammatory response (61, 62). Gai et al. found that there was an imbalance of flora 12 hours after CLP in the mouse model, with significant reductions in Firmicutes and Bacteroidetes (63). Giridharan et al. found that acetate, propionate and butyrate were significantly down-regulated in the CLP model group compared with the control group (55). Thus, disruption of gut microbiota during sepsis leads to secondary down-regulation of SCFAs levels. Meanwhile, SCFAs, metabolites of the gut microbiota, could reshape intestinal dysbiosis, reduce the release of inflammatory cytokines, and improve survival rates in CLP-induced model (54). Therefore, the stability of gut microbiota is crucial for the production of SCFAs and host immune regulation. Meanwhile, SCFAs as products in turn maintain the intestinal ecological balance and participate in the regulation of immune response.

Acetate, propionate, and butyrate are the primary SCFAs found in the gut, accounting for more than 95% of SCFAs, of the total SCFAs composition, with an estimated ratio of approximately 3:1:1 (64). The relative abundance of these SCFAs varies depending on factors such as the host’s diet, microbiome composition, and tissue location, as they are produced by specific microbial communities in different regions of the gut (65). Acetate, the most abundant SCFAs, is extensively produced in the gut and can be metabolized and transported by the intestinal epithelium and liver, serving as an energy source (66). Gut bacteria can convert pyruvate to acetate by either acetyl-CoA or the Wood-Ljungdahl pathway (67, 68). The production of propionate mainly occurs in the large intestine and is produced by bacteria such as Bacteroides and Clostridium anaerobes (69). In addition, the succinate pathway is an important pathway for the production of propionic acid by human gut microbiota, and methylmalonyl-CoA is the key enzyme in the succinate pathway. Propionic acid can also be generated by the acrylate pathway and the propylene glycol pathway (70, 71). Propionate can be absorbed by the intestinal epithelium and transported to the liver, where it is further metabolized into glucose or fatty acids (72). Butyrate is formed by condensation and subsequent reduction of two acetyl-CoA molecules to butyrate, which can be converted to butyrate by phospho-transbutyrylase and butyrate kinase (73). Butyrate can be absorbed by intestinal epithelial cells and metabolized in the liver as ketone bodies or carbon dioxide (74). Studies have shown that the concentration of SCFAs in the intestine ranges from 20 to 140 mM, with extremely high concentrations in the proximal colon (70 to 140 mM), and relatively low concentrations in the distal colon (20 to 70 mM) and distal ileum (20 to 40 mM), depending on the presence of infection/inflammation in the host (32, 75). In addition, studies have found that the production of gut-derived SCFAs can be regulated by changing the proportion of SCFAs -producing flora in the gut (76). At present, the main ways to regulate the structure of gut microbiota include changing the host dietary structure (Mediterranean diet), direct intake of probiotics or prebiotics to regulate gut microbiota (77–79). In conclusion, gut microbiota plays a crucial role in the generation of SCFAs.

As the most abundant metabolite of the intestinal microbiota in the intestinal lumen, SCFAs play a multifaceted role in the regulation of the host immune system (80). Firstly, SCFAs serve as a vital energy source for colon and ileal cells, influencing the expression of genes involved in intestinal epithelial barrier function and defense mechanisms (32). Second, modulate the function of key immune cells such as macrophages, neutrophils, dendritic cells, and even adipocytes, thereby impacting inflammation progression through the regulation of TNF-α, IL-10, and IL-4 levels in adipose tissue (81). Thirdly, SCFAs exert their immunomodulatory effects by inhibiting HDAC activity and activating specific receptors, namely, free fatty acid receptors type 2 and 3 (FFA2 and FFA3 receptors) and G protein-coupled receptor 109A (GPR109A) (82). These mechanisms collectively contribute to the regulation of immune responses and the formation of various post-translational modifications involved in cellular processes.

## 4 The relationship between sepsis and SCFAs metabolism

### 4.1 Changes in SCFAs levels in sepsis and their relationship to disease progression or regression

The pivotal pathway responsible for the production of SCFAs has been found to be intricately linked with the presence of microbiota (80). Notably, a recent study has demonstrated that individuals with diminished gut microbiota diversity face an elevated susceptibility to sepsis (83, 84). Furthermore, in an observational investigation involving critically ill patients with non-abdominal infections, perturbations in the gut microbiome have been shown to predispose individuals to sepsis by enabling the proliferation of pathogens, promoting an aberrant immune response, and impeding the production of beneficial SCFAs (85). Additionally, the concentration of propionic acid in serum has been found to increase in tandem with the severity of sepsis, suggesting that serum propionate holds significant potential as a predictor and prognostic biomarker for septic patients (86). Consequently, alterations in SCFAs levels during sepsis may result from an imbalance in intestinal flora, thereby inducing immune dysregulation and damage to the intestinal epithelial barrier. This, in turn, exacerbates the detrimental cycle of immune dysregulation prompted by the inflammatory response to sepsis.

Furthermore, Liao et al. have observed a significant downregulation of acetic acid and propionic acid in the CLP-induced model relative to the sham group. Remarkably, exogenous supplementation of SCFAs has been shown to substantially increase acetic acid and propionic acid levels while significantly downregulating the levels of IL-1β, IL-6, and TNF-α (38). Similarly, Li et al. have discovered that in mice subjected to the CLP model, SCFAs levels are significantly reduced, accompanied by considerable alterations in the gut microbiota. Notably, exogenous SCFAs administration has been found to enhance intestinal barrier integrity and significantly downregulate IL-1β, TNF-α, and IL-6 levels in mice (87). Although SCFAs may have potential value in the prevention and treatment of sepsis, its feasibility for the treatment of clinical patients should be verified by human trials.

### 4.2 The regulatory effect of SCFAs on sepsis

SCFAs exert multiple modulatory effects on the pathophysiology of sepsis. First, SCFAs are a key source of energy for colon and ileal cells and affect intestinal epithelial barrier and defense functions by regulating related gene expression (68). Wang et al. found that butyrate provides energy to colonic intestinal epithelial cells, regulates intestinal gene expression, inhibits the intestinal inflammation induced by LPS (88). Zhan et al. have demonstrated that SCFAs hinder pathogen invasion by supplying energy and regulating the barrier function and immune status of the host intestine (89). Secondly, SCFAs intervene in the inflammatory storm phase during sepsis by regulating the production of immune cytokines (**Table 1**). At present, a number of studies have found that SCFAs can significantly down-regulate the levels of a variety of pro-inflammatory mediators (38, 90, 92, 93). For example, Vinolo et al. found that butyrate and propionate can reduce the expression of TNF-α and nitric oxide synthase (NOS) in primary mouse neutrophils induced by LPS (56). Similarly, Wang et al. demonstrated that butyrate significantly decreased the levels of TNF-α, IL-1β and IL-6 in LPS-injected mice (91). In another study, butyrate treatment was shown to inhibit the levels of nitric oxide, IL-6, and IL-12 inflammatory mediators in LPS-stimulated bone marrow-derived macrophage (BMDM) cells by inhibiting HDAC (7). Thirdly, SCFAs could regulate sepsis by regulating PTMs. For example, in CD8^+^ T cells within a low-glucose tumor environment, acetate facilitates histone acetylation to enhance the transcription of the IFN-γ gene and cytokine production, thereby modulating the progression of inflammation (94). In addition, acetate can supplement acetyl-CoA, which promotes the acetylation of GAPDH to enhance its activity, thereby promoting glycolysis, so as to promote rapid memory CD8(+) T cell response and enhance the immune response (95). Taken together, these findings suggest that SCFAs can suppress the inflammatory response, and that the absence of SCFAs and the reaction of SCFAs with PTMs are secondary events of the inflammatory response in sepsis.

**Table 1:** SCFAs regulate inflammation by regulating the production of immune cytokines.

SCFAs	Model	Pro-/anti-inflammatory	Signaling pathway	Ref
Acetate	LPS-induced	anti-	TNF-α↓	(90)
Butyrate	LPS-induced	anti-	CO↓, IL-6↓, IL-12↓	(7)
Butyrate	LPS-induced	anti-	IL-6↓, TNF-α↓, IL-1β↓, IL-10↑	(91)
Propionate	LPS-induced	anti-	iNOS↓, NF-kB↓, COX-2↓	(92)
Butyrate	CLP-induced	anti-	iNOS↓, COX-2↓	(59)
Acetate Propionate Butyrate	CLP-induced	anti-	IL-6↓, TNF-α↓, IL-1β↓	(38)
Acetate Propionate Butyrate	CLP-induced	anti-	IL-18↓, NLRP3↓, IL-1β↓	(54)
Butyrate	CLP-induced	anti-	NF-kB↓, IL-6↓, TNF-α↓, IL-1β↓	(93)

The symbols "↑" represent increase. The symbols "↓" represent decrease.

### 4.3 The mechanism of SCFAs regulating post-translational modification of proteins in sepsis

PTMs represent a crucial pathway for regulating protein function, and significant changes in phosphorylation, ubiquitination, methylation, and acylation have been observed during and after sepsis (96–98). Lysine acylation includes long chain acylation and short chain acylation. Protein acylation is involved in a variety of cellular processes, such as protein stability, protein subcellular localization, enzyme activity, transcriptional activity, protein-protein interaction and protein-DNA interaction (99). Among them, protein lysine acylation exerts important and unique regulatory roles. Studies have indicated that lysine acylation can regulate the development of sepsis by regulating the activity of modifying enzymes, targeting innate sensors and downstream signaling molecules (100, 101).

Interestingly, the most substrates of these novel lysine acylation modification are SCFAs, such as acetate, malonate, crotonic acid and 2-hydroxyisobutyric acid, among which lysine acetylation (Kac), lysine malonylation (Kmal), lysine crotonylation (Kcr) and lysine 2-hydroxyisobutyrylation (Khib) play crucial roles in inflammation and immunity. Consequently, SCFAs may participate in regulating the immune response during sepsis by influencing the corresponding acylation modification levels (102–105). To simplify the intricate regulation of SCFAs-mediated PTMs on the immune system, this review will combine current research to elucidate the mechanism by which SCFAs affect PTMs through two primary pathways: i: SCFAs as inhibitors of HDAC that regulate the level of lysine acylation modification and participate in the regulation of sepsis; ii. SCFAs as substrates for lysine post-translational modification that regulate the level of modification and participate in the regulation of sepsis.

#### 4.3.1 SCFAs as inhibitors of HDAC

##### 4.3.1.1 Methylation

Protein lysine methylation is a dynamic process that plays a vital role in various biological processes, such as DNA damage repair, cell growth, metabolism, and signal transduction (106). The dynamic lysine methylation system comprises three key components: protein lysine methyltransferases (PKMTs), which add methylation marks; methyl-binding domains (MBDs), which participate in methylation events with biological outcomes; and lysine-specific demethylases (KDMs), responsible for the removal of methylation marks (107).

In the context of early sepsis, protein lysine methylation assumes a critical role in the epigenetic regulation of innate immunity. Xia et al. discovered that Ash1l, an H3K4 methyltransferase, suppressed the expression of TNF-α and IL-6 by promoting H3K4 methylation at the Tnfaip3 promoter in mice injected with LPS (108). Interestingly, SCFAs also regulate inflammation progression and immune cell functional expression by modulating lysine methylation modifications. Kaye et al. observed that in hypertensive mice, acetate activated DNA methylation in Treg cell regions, enhancing the anti-inflammatory effects of immune cells by regulating Treg cell proliferation (109). Additionally, Chang et al. found that in LPS-stimulated BMDM cells, exogenous addition of butyrate could inhibit the methylation modification of the GPR41/43 promoter region, thereby suppressing the expression of GPR41/43 and reducing host inflammatory damage (7). Collectively, the methylation of histone and non-histone lysine significantly influences the function of immune cells during sepsis. SCFAs regulate the levels of inflammatory and anti-inflammatory factors by inhibiting HDAC activity, thereby affecting methylation levels and interfering with the transcriptional activities of transcription factors. Hence, SCFAs hold promise as potential therapeutic agents for early sepsis treatment.

##### 4.3.1.2 Acetylation

Lysine acetylation modifications are a universally recognized group of PTMs that that have the potential to impact protein function through various mechanisms. These mechanisms include the regulation of protein stability, enzymatic activity, subcellular localization, interplay with other post-translational modifications, and the control of protein-protein and protein-DNA interactions(110). Among these, the diverse HAT/KAT and HDAC/KDAC enzymes are widely involved in biological cellular processes, and the lysine acetylation or deacetylation they induce in cells may contribute to the development of several diseases.

SCFAs play a role in the regulation of lysine acetylation modifications by inhibiting HDAC activity. This interference with HDAC activity has been shown to impact the progression of inflammation in various diseases (111–118). Notably, Luu et al. discovered that in experimental mouse models of colitis, valerate promotes the acetylation of histone H4 at the IL-10 promoter by providing acetyl-CoA and inhibiting HDAC activity. This, in turn, induces the production of IL-10 in lymphocytes, playing an anti-inflammatory immunomodulatory role (119). Moreover, Park et al. found that acetate upregulates the acetylation modification of p70 S6 kinase by inhibiting HDAC activity. This promotes the differentiation of T cells into effector T cells and regulatory T cells, consequently reducing anti-CD3-induced inflammation in an IL-10-dependent manner (120). Meanwhile, Yang et al. discovered that in anti-CD3-activated CD4^+^ cells, butyrate upregulates histone acetylation on the IL-22 promoter, facilitating HIF1α binding to this region. This ultimately promotes the production of IL-22, safeguarding the host from inflammation (121). Therefore, SCFAs can regulate inflammatory and immune processes by regulating protein acetylation modification, which may potentially exert a similar effect on inhibiting hyperinflammation in sepsis. In conclusion, facilitate the acetylation of both histone and non-histone proteins by inhibiting HDAC activity. This regulation of lysine acetylation contributes to the innate immune response of the host and participates in the immune pathogenesis of sepsis. Consequently, targeting key lysine acetylation sites to inhibit inflammation progression represents an important approach to the treatment and intervention of sepsis.

##### 4.3.1.3 Crotonylation

Lysine crotonylation (Kcr) modification is a highly conserved protein modification that has evolved from histone lysine acetylation (Kac). Although crotonylation and acetylation have distinct biological characteristics, they share the same regulatory enzymes. Crotonylation modification plays a role in regulating diverse biological processes and the development of various diseases, including gene expression, spermatogenesis, cell cycle regulation, and the pathogenesis of conditions ranging from depression to cancer (122–125). In addition to intracellular crotonyl-CoA, the regulation of Kcr also involves the addition and removal of modified writers and erasers, respectively, contributing to homeostasis (126). Histone acetyltransferases (HATs), such as p300/CBP, possess significant histone crotonyl transferase (HCT) activity, highlighting their role in crotonylation modification (127).

It has been also demonstrated that crotonylation is involved in sepsis. Sabari et al. found that crotonyl-CoA supplementation in macrophages led to a substantial increase in histone H3K18Cr. Additionally, knockdown of ACSS2, an enzyme involved in crotonyl-CoA synthesis, reduced the expression of histone H3K18Cr and inflammatory genes in LPS-stimulated RAW264.7 cells (128). This suggests a close association between crotonylation and sepsis, with the possibility of modulating inflammatory progression through the regulation of crotonylation. Furthermore, SCFAs are involved in regulating Kcr modification levels by inhibiting HDAC activity. Fellows et al. demonstrated that exogenous butyrate supplementation inhibited HDAC activity in colonic epithelial cells, resulting in the upregulation of histone H3K18cr modification and influencing the cell cycle. This finding further underscores the connection between SCFAs and chromatin signaling (129). Collectively, the levels of lysine Kcr modification undergo significant changes following LPS stimulation, and SCFAs participate in the regulation of Kcr by inhibiting HDAC activity. This suggests that SCFAs may impact sepsis through the modulation of Kcr modification. Therefore, it is crucial to further investigate the mechanisms underlying histone and non-histone Kcr modification targets in sepsis for the development of effective treatments.

#### 4.3.2 SCFAs as substrates for lysine acylation modification

##### 4.3.2.1 Lysine 2-hydroxyisobutyrylation

Lysine 2-hydroxyisobutyrylation (Khib) is a novel histone acylation modification first identified in 2014 with 2-hydroxybutyrate and its cozymoylated form 2-hydroxyisobutyl CoA as the substrates (130–132). The 2-hydroxybutyric acid is a typical SCFAs detected in micromolar concentrations in a variety of human biological fluids. Of note, its high occurrence in the urine of obese patients was found associated with the abundance of specific taxa of the gut microbiota (133, 134). Similar to other types of PTMs, khib also maintains a dynamic balance by adding and removing writers and erasers respectively. Huang et al. demonstrated that Esa1p and its human homolog TIP60 from yeast cells can regulate khib proteinogenesis and act as writers for khib (130). Additionally, the lysine acetyltransferase p300, mentioned earlier, exhibits enzymatic activity not only for lysine acetylation but also for lysine butyrylation, propionylation, and crotonylation (135–137a).

Although khib occurs in diverse cellular proteins, it has only emerged to realize that it may exert mutifaced roles physiological and pathological conditions. For instance, Yamazaki et al. found that the serum level of 2-HIBA was significantly up-regulated in a mouse model of periodontitis through metabolomics (138). Similarly, Tsoukalas et al. found statistically significant differences in the metabolite levels of 2-hydroxyisobutyric acid in urine of rheumatoid arthritis patients through metabolomics (139). Therefore, as a SCFAs, the level of 2-hydroxyisobutyric acid is significantly up-regulated in a variety of diseases involving inflammation, suggesting that the change of its level has an important relationship with the development of inflammation. Notably, the occurrence of khib is also strongly associated with inflammatory regulation. Ge et al. found that in the skin tissue of patients with psoriasis, the proteins encoded by S100A9, FUBP1 and SERPINB2 genes have significant khib with proteins in the PI3K-Akt signaling pathway (137b). Among them, S100A9 is a potential target for the treatment of sepsis, and further investigation is needed to determine if its khib is involved in sepsis progression (140). In addition, Dong et al. analyzed khib occurrence in key enzymes of the glycolytic pathway in blood monocytes from end-stage renal disease patients, which may affect immune cell numbers and induce immune senescence by influencing glycolytic function (141). Furthermore, Xie et al. found high enrichment of khib in blood monocytes from lupus erythematosus patients during the antigen processing and presentation process and leukocyte migration pathways. This suggests that khib is involved in inflammation regulation by affecting the khib level of key proteins in these pathways (142). Although there is no direct reported relationship between khib and sepsis, khib is essentially involved in the regulation of various inflammation-related diseases, implicating the necessity to further elucidate the specific mechanism of immune function regulated by khib-modified histone or non-histone proteins and discover new targets for sepsis treatment.

##### 4.3.2.2 Lysine malonylation

Lysine malonylation (Kmal) is a highly conserved acylation modification that was initially discovered in 2011 (143). Its modified substrates include malonate and malonyl-CoA. The occurrence of Kmal relies on the addition of malonyl groups to lysine by malonyl-CoA, resulting in its charge changed from +1 to -1. Of note, malonate is also a typical SCFAs which participates in the regulation of mammalian glycolytic physiological processes (144, 145). Similar to other acylation modifications, the extent of Kmal modification is governed by the de-modifying enzyme. For example, Nishida et al. unveiled that SIRT5 functions as a global regulator of lysine malonylation and orchestrates the energy cycle through the glycolytic pathway (146).

Kmal is also implicated in regulating intracellular inflammatory and immune events. In a study conducted by Lee et al., it was discovered that malonate profoundly hindered the activation of the p38 MAPK/NF-κB pathway in LPS-stimulated microglia, thereby exerting an anti-inflammatory effect (147). Additionally, Park et al. observed that malonate significantly downregulated the expression of ROS-induced NF-κB and inflammation-related cytokines (IL-6, COX-2, and TNF-α) in HaCaT cells(148). Hence, it is plausible that malonate, acting as a SCFAs, modulates the innate immune response through the inhibition of the inflammatory cascade. Interestingly, Galván-Peña et al. demonstrated that the level of Kmal was significantly upregulated in LPS-stimulated BMDM cells. Notably, malonylation mass spectrometry revealed the presence of malonylation modifications at the lysine 213 site on GAPDH. Under normal circumstances, GAPDH binds to and represses the translation of various mRNAs associated with inflammation, including the one encoding TNFα. However, upon malonylation modification of GAPDH, it dissociates from the TNFα mRNA, thereby promoting its translation (149). Furthermore, Qu et al. (150) found that atractylodin inhibits the malonylation of GAPDH and subsequently suppresses the level of TNF-α in LPS-induced RAW264.7 cells. Consequently, Kmal modification may play a pivotal role in the regulation of sepsis. The identification of novel targets of Kmal-modified histones or non-histones holds great significance for the treatment of sepsis.

**Figure 1:**
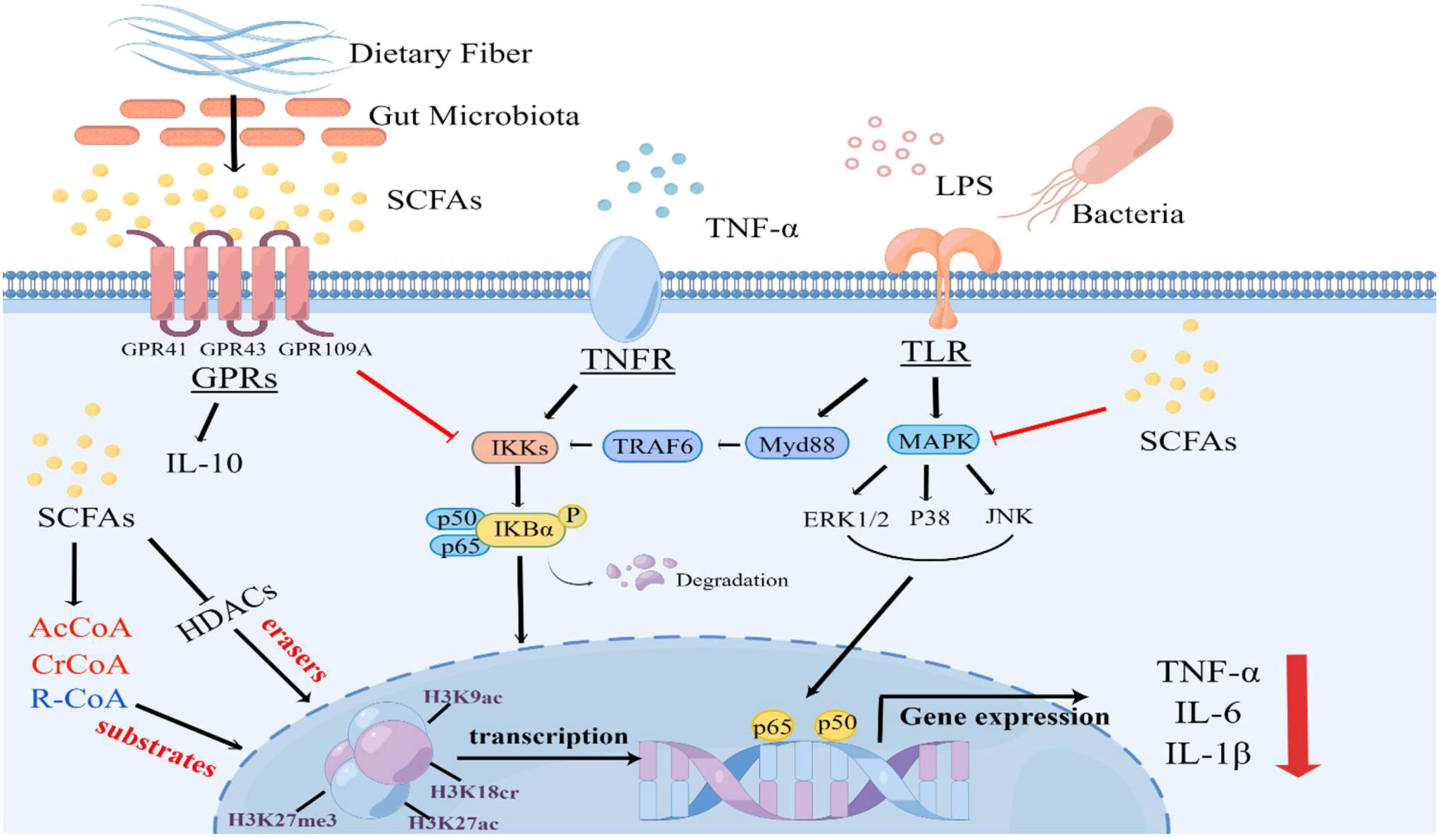
An overview on the effects of SCFAs on signaling pathways and protein post-translational modifications during LPS or TNFα-induced sepsis.

**Figure 1** An overview on the effects of SCFAs on signaling pathways and protein post-translational modifications during sepsis. The binding of LPS or TNF-α to their respective receptors triggers the activation of Toll-like receptor 4 (TLR4), MAPK and nuclear factor kappa B (NF-κB) signaling pathways, leading to the release of cytokines, which plays a crucial role in sepsis development. However, SCFAs, predominantly acetate, propionate, and butyrate, exert anti-inflammatory effects by activating G protein-coupled receptor (GPR) 41, GPR43, and GPR109A receptors or inhibiting the activation of NF-κB and mitogen-activated protein kinase (MAPK) signaling pathways. Furthermore, SCFAs can serve as substrates for protein post-translational modifications and inhibit the activity of HDAC, thereby regulating the levels of various lysine acylation modifications. This inhibition of HDAC activity leads to the transcriptional downregulation of inflammatory factors and actively participates in immune regulation within the body (By Figdraw).

## 5 Summary and perspectives

As a byproduct of intestinal bacteria and a metabolite, SCFAs actively participate in the regulation of the host’s immune function, playing a crucial role in sepsis and inflammatory diseases. Furthermore, PTMs are closely associated with key stages of sepsis, exerting their influence on the regulation of cytokine storms and immune suppression by inhibiting or reversing the transcription of pro-inflammatory genes. Interestingly, both the epigenetic changes caused by PTMs and the immunosuppressive state of sepsis are long-lasting conditions. In our review, we have focused on the role of SCFAs, which may have a significant impact on the regulation of lysine acylation modifications in sepsis. There is mounting evidence suggesting that SCFAs can modulate innate and adaptive immune responses by regulating lysine acylation modifications (**Table 2**).

**Table 2:** Regulation of protein post-translational modifications by SCFAs in sepsis.

SCFAs	Histone/non-histone	PTMs changes	Model	Roles	Ref
Acetate	NIP45	Methylation	T-cell	Acetate alters DNA methylation in the region of activated Treg cells, regulates Treg cell proliferation and enhances the anti-inflammatory effect of immune cells.	(109)
Acetate	Foxp3 H3K9ac	Acetylation	T-cell	Acetate may increase acetylation of the Foxp3 promoter through HDAC9 inhibition by increasing the number and function of T regulatory cells and significant airway inflammatory responses.	(117)
Acetate	p70-S6K	Acetylation	T-cell	The inhibitory effect of SCFA on HDAC in T-cells increases acetylation of p70 S6 kinase and promotes T-cell differentiation into effector and regulatory T-cells, thereby promoting immunity or immune tolerance depending on the immune environment.	(120)
propionate	H3K9ac	Acetylation	cTregs	Propionic acid treatment of cTregs enhanced histone H3K9ac through inhibition of HDAC, which in turn affected the immune status of cTregs.	(115)
Pentanoate	H4	Acetylation	Th17 cell	Pentanoate promotes histone H4 acetylation by providing acetyl coenzyme A and inhibiting the activity of HDAC, induces IL-10 production by lymphocytes by increasing acetylation at the IL-10 promoter, and improves anti-inflammatory capacity.	(119)
Butyrate	H3	Acetylation	CD4^+^ T cells	Exogenous addition of butyrate enhances histone H3 acetylation in the promoter and conserved non-coding sequence regions of the Foxp3 locus, induces differentiation of mouse colonic T-cells and ameliorates the development of colitis.	(118)
Butyrate	H3K9ac/ H3K9me3	Acetylation	CD4^+^ T cells	Butyrate protects the host from inflammation by increasing the binding of HIF1α to the IL-22 promoter through histone modifications and promoting IL-22 production.	(121)
Butyrate	H3K9ac	Acetylation	BMDM	Butyrate maintains tolerance to intestinal microbiota by inhibiting the activity of HDAC, promoting Nos2, IL-6 and H3K9ac, the promoter region of the IL12b gene, and by down-regulating the release of pro-inflammatory factors that reduce the response of macrophages to commensal bacteria.	(7)
Butyrate	GPR41/ GPR43	Methylation	Cecal Tissues	Butyrate inhibits the methylation of the GPR41/43 promoter region and thus the expression of GPR41/43, reducing inflammatory damage.	(103)
Butyrate	H3K18cr	Crotonylation	Colon epithelial cell	Butyrate promotes histone crotonylation by inhibiting the activity of HDAC in colonic epithelial cells.	(129)
Butyrate/ Propionate	Foxp3	Acetylation	peripheral regulatory T-cell	Butyrate and propionate increase acetylation of Foxp3 to promote extrathymic Treg cell differentiation by inhibiting HDAC activity.	(104)
Butyrate/ Propionate	H4K12ac	Acetylation	BMDM	Treatment of bone marrow cells with butyrate and propionate resulted in upregulation of H4K12ac on the PU.1 promoter, inhibiting the expression of PU.1 and RelB and thus preventing DC development.	(111)
isobutyric/ propionic	H3K27ac/ H3K27me3	Acetylation	T-cell	SCFAs regulates host antiviral innate immune response by increasing histone acetylation and decreasing repressive histone methylation to coordinate viral transcriptional activation.	(116)

In this review, we have summarized the research progress regarding SCFAs in the pathological processes of inflammation and sepsis by their impact on PTMs. While host metabolism and immune regulation have garnered significant attention, further experiments are required to elucidate the specific mechanisms through which SCFAs regulate different lysine acylation modifications and the interplay between these modifications in sepsis-induced inflammation and immune suppression. Building upon the current research progress, we propose a scientific hypothesis that exogenous supplementation of SCFAs can regulate the levels of lysine acylation modifications, thereby restoring the immune response function of host immune cells and reversing host immunosuppression. With the advancement of metabolomics and proteomics technologies, it becomes increasingly feasible to explore the molecular targets of different lysine acylation modifications regulated by SCFAs during the immunosuppression stage of sepsis. Consequently, targeting lysine acylation regulated by SCFAs emerges as a potential therapeutic strategy for treating immunosuppression in sepsis, although further clinical trials are necessary to validate its feasibility in the future.

## Author contributions

LZ and XS collected documents, wrote the manuscript and drew the mechanism figures. HQ collected documents and made tables. SL and TY polished the language and revised the manuscript. XLL and XL conceived ideas, designed the structure of the manuscript and revised the manuscript. All authors read and approved the submitted version. All authors contributed to the article.
